# Sex-Specific Environmental Impacts on Initiation and Progression of Multiple Sclerosis

**DOI:** 10.3389/fneur.2022.835162

**Published:** 2022-02-03

**Authors:** Jonatan Leffler, Stephanie Trend, Shelley Gorman, Prue H. Hart

**Affiliations:** ^1^Telethon Kids Institute, University of Western Australia, Nedlands, WA, Australia; ^2^Centre for Neuromuscular and Neurological Disorders, Perron Institute for Neurological and Translational Science, University of Western Australia, Perth, WA, Australia

**Keywords:** multiple sclerosis, sex hormones, environmental risk factors, immune regulation, Epstein-Barr virus (EBV), UV radiation, vitamin D

## Abstract

The immunological mechanisms that contribute to multiple sclerosis (MS) differ between males and females. Females are 2–3 times more likely to develop MS compared to males, however the reason for this discrepancy is unknown. Once MS is established, there is a more inflammatory yet milder form of disease in females whereas males generally suffer from more severe disease and faster progression, neural degradation, and disability. Some of these differences relate to genetics, including genetic control of immune regulatory genes on the X-chromosome, as well as immune modulatory properties of sex hormones. Differences in MS development may also relate to how sex interacts with environmental risk factors. There are several environmental risk factors for MS including late-onset Epstein Barr virus infection, low serum vitamin D levels, low UV radiation exposure, smoking, obesity, and lack of physical activity. Most of these risk factors impact males and females differently, either due to biological or immunological processes or through behavioral differences. In this review, we explore these differences further and focus on how the interaction of environmental risk factors with sex hormones may contribute to significantly different prevalence and pathology of MS in males and females.

## Introduction

**Figure 1:**
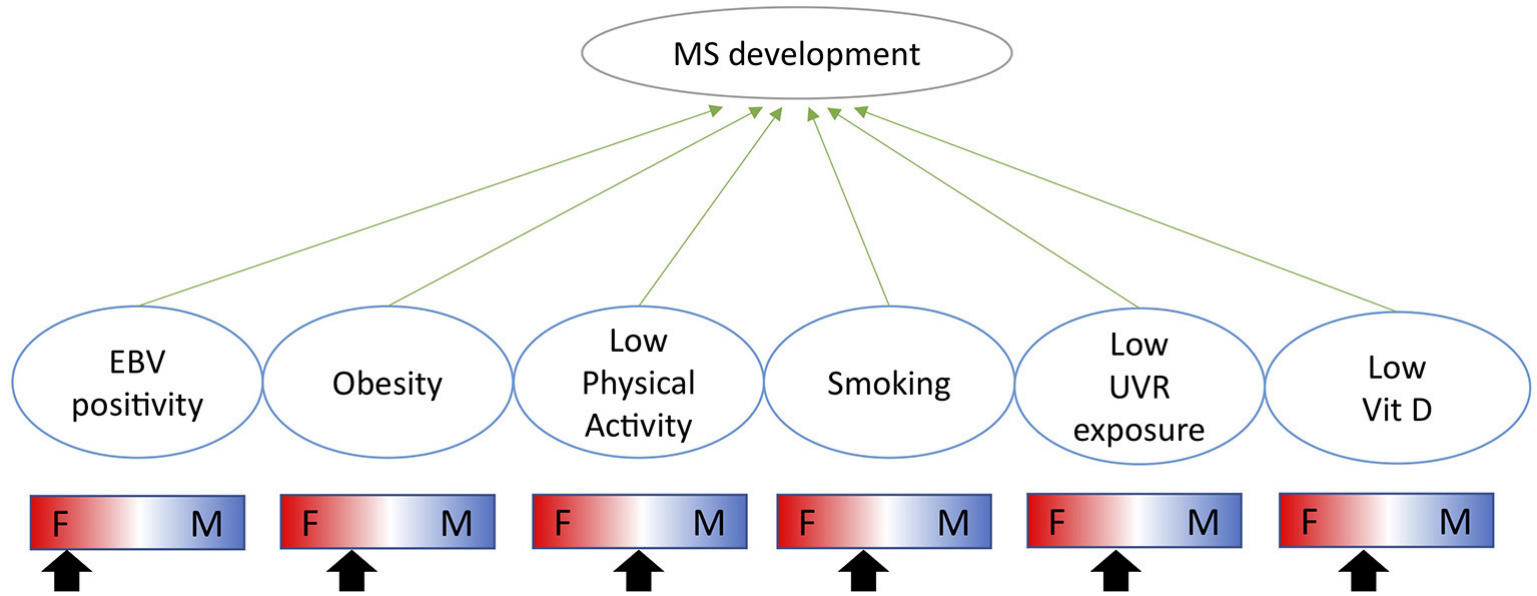
Indication of how each environmental risk factor impacts males and females differently and whether each factor is likely to increase the risk of MS more in females (F) or males (M) as indicated by the arrow.

The immunological impact of biological sex is highlighted by significantly different prevalences of immune mediated diseases between males and females. Males are more susceptible to infectious diseases, whereas females are more susceptible to autoimmune diseases such as multiple sclerosis (MS) and systemic lupus erythematosus (SLE) (1). The impact of sex on disease prevalence stems from both genetic and environmental factors as well as their interaction. Genetically, several immunologically important genes are expressed from the X-chromosome such as TLR7 and FoxP3 (2). Environmentally, sex hormones are potent immune modulators and exert their function through binding to extra- and intra-cellular receptors, widely expressed across the immune system. Sex differences in responses to environmental cues may influence the initiation and/or maintenance of autoimmune diseases. The genetic differences in how the immune system operates have been reviewed in detail elsewhere (1) as well as in the context of MS (3). This review will focus on the specific impact of sex hormones on the immune system and their interaction with environmental risk factors. The sex-specific impact of each environmental factor is summarized in (Figure 1).

## Multiple Sclerosis

MS is a chronic inflammatory and neurodegenerative disease resulting in demyelination of neuron fibers, inefficient signal transmission and reduced muscular mobility. MS affects approximately 30 in 100,000 globally (4) and ~100 in 100,000 individuals in Australia (5). Prevalence of MS has increased over the last decades, particularly in females (6) who are now 2–3 times more likely to develop MS than males (6, 7). At the beginning of the century, prevalence was estimated to be proportional between males and females (8), suggesting that changes to environmental factors likely have impacted MS development during the last decades.

There are two forms of MS. The majority of individuals develop remittent relapsing MS (RRMS) where periods of disease activity (flare-ups) are followed by periods of low-symptomatic activity or absence of symptoms (remissions) (9). In 10–15% of patients, primary progressive MS is established and results in faster deterioration with no episodes of remission. RRMS is significantly more common in females compared to males, whereas there is less difference in incidence for primary progressive MS (10). In RRMS, females display elevated levels of inflammation in the CNS compared to males (11) whereas males display signs of more severe neurodegeneration and more disabling manifestations (11, 12). Over time RRMS converts into secondary progressive MS where episodes of remission cease; this appears to occur faster in males compared to females (12). As the disease progresses, MS leads to a debilitating lack of muscular mobility and cognitive deterioration. The main therapies involve disease modifying drugs including interferon (IFN)-β and biologicals such as B cell depleting monoclonals. Available treatments can slow disease progression, but no treatment is yet able to prevent or reverse disease progression.

There are several risk factors for MS, including genetic polymorphisms, relating mainly to variants in HLA-DR, where HLA-DRB1^*^15:01 has the strongest link with increased risk of MS (13). There are also several established environmental risk factors including Epstein-Barr virus (EBV) infection, low UV radiation (UVR) exposure, vitamin D deficiency, smoking, obesity and reduced physical exercise (14–16). How these impact MS risk and pathology in male and females will be discussed below.

### Immune Mechanisms of MS

Traditionally, MS is viewed as a T cell dependent disease, particularly driven by activated and clonally expanded CD8^+^ T cells that are found in CNS lesions. These are likely activated in the periphery, potentially by an underlying viral infection such as Epstein-Barr virus (EBV). Target antigens in the CNS remain elusive but T cells specific for myelin-associated proteins are regularly observed (17). CD4^+^ T cells, mainly Th1, Th17 and Tregs, are also involved in MS pathology as recently reviewed (18). Their involvement likely explains the strong association of MS and HLA-DR haplotypes (19). MS lesions also contain a considerable proportion of B cells and monocytes. The role of the B cells in MS is still emerging (20). Increased IgGs are commonly observed in CSF from MS patients, however their contribution to MS pathology remain unclear (21). Instead, antibody-independent functions of B cells, such as antigen presentation or cytokine production may be more relevant (22). Evidence to support this stems from observations that MS patients benefit rapidly from anti-CD20 therapy that depletes several B cell subsets except antibody-producing plasma cells that lack CD20 expression (23).

## Impact of Sex Hormones on the Immune System

The contribution of sex hormones during the development of MS remains unclear (24). Sex hormones have several distinct effects across the immune system that are relevant for MS pathology. Estrogen can promote either Th1 (25) or Th2 (26) differentiation at low or high concentrations, respectively. Estrogen may also expand FoxP3^+^ Tregs (27) while suppressing Th17 cells as demonstrated in an EAE model (28). Estrogen is essential for generation of inflammatory DC (29) and may interfere with B cell selection allowing for the escape of autoreactive B cells (30). Progesterone appears to prevent inflammation through reducing the activation of DC (31, 32), and inhibits Th1 and Th17 differentiation (33) whilst promoting Th2 immunity at high concentrations (34). This is likely important for fetus tolerance during pregnancy. Testosterone has general anti-inflammatory effects including dampening of macrophage and DC activation (35) and inhibition of Th1 immune differentiation (36, 37).

### Impact by Sex Hormones on MS Pathology

In MS, the female sex hormones, estrogen and progesterone, appear to have protective effects; this is surprising given the increased prevalence of MS in females. However, this is supported by low risk of relapse during pregnancy (38) and the subsequent increased risk of relapse once pregnancy hormones return to normal (38). Decreased levels of estrogen are also associated with disease relapse in female patients (39). The impact of oral contraceptives remains unclear (40). Attempts to reduce MS symptoms through administration of estrogen derivates have shown some promise (41, 42), however further studies are required. Progesterone has been suggested to promote myelination in the CNS (43, 44) however does not appear to have an effect on relapse rate as reviewed (45). Not all female specific hormones may be beneficial in MS as exemplified by prolactin which is increased in female MS patients and promotes formation of autoreactive B cells (46). In male MS patients, up to 40% display decreased levels of testosterone compared to matched controls (47). Pilot studies further suggest that testosterone may ameliorate MS symptoms in males (48), however the mechanisms remain unclear but are likely mediated through the immune regulatory properties of testosterone as described above.

Given the importance of sex hormones in MS pathology, transgender individuals who take sex hormones to align their body with their gender may be subject to potential changes in risk of MS and disease pathology. Although this is a neglected area of research, a clinical records-based study identified an increased incidence of MS in trans individuals who were assigned as males at birth (49). These individuals are likely to be on estrogen supplementation or anti-testosterone treatment. No change in risk was observed for trans individuals who were assigned as females at birth and likely received testosterone treatment.

Together, these observations suggest that although the main female sex hormones promote immune activation and females are at increased risk of developing MS, female sex hormones may still have a beneficial impact on MS pathology. Whether the increased prevalence of MS in females instead relates to differences in hormone receptor expression, genetic differences related to the X-chromosome or sex-specific mechanisms in the CNS, such as increased ability of microglia in females to induce inflammation in response to cell damage, remains to be explored (3). How sex hormones interact with environmental risk factors are discussed below.

## Impact of Environmental Risk Factors on MS Risk in Males and Females

### Do Female-Specific Viral Responses Contribute to Skewed MS Prevalence?

Epstein-Barr virus (EBV) causes a chronic infection of B cells, that reprograms resting B cells into a memory-like phenotype. Over 95% of the adult population have serological evidence of past EBV infection (50). EBV is associated with development of several autoimmune diseases and B cell lymphomas (51). It is one of the strongest environmental risk factors for MS development. If EBV is acquired during childhood, the risk of subsequent MS development is low. However, if EBV is acquired during adolescence or early adulthood, there is a significantly increased risk of MS development, particularly if the individual experiences severe disease or hospitalization (52, 53). Severity and EBV-specific antibody titres correlate directly to risk of MS development (54). Modeling suggests that the difference in risk between childhood and adolescence is related to puberty onset, pointing toward a role for sex hormones (55). Females display elevated levels of EBV reactive antibodies compared to males also suggesting a more robust EBV response in females (56, 57). Plasmacytoid DC (pDC) from females typically produce more type I IFN following virus exposure, particularly through TLR7 (58), which together with TLR9 (59) recognizes EBV RNA and DNA in infected cells (60). Type I IFN production is also directly regulated by estrogen and associated with serum testosterone levels (61, 62). EBV-induced type I IFN production is thought to contribute to SLE development (60), whereas in MS, the ability of pDC to produce type I IFN is reduced (63, 64). Interferon-β treatment is successfully used to reduce relapse frequency and delay neurological disability in MS (65, 66). Whether reduced type I IFN responsiveness is an endogenous deficiency in MS that predisposes an individual to more severe EBV infection or if it relates to EBV's ability to inhibit type I IFN signaling as a mechanism for avoiding the immune system (67) remains to be elucidated.

### The Impact of BMI and Physical Activity on MS Risk in Males and Females

Obesity during childhood/adolescence (68, 69) or adulthood (70) as well as reduced physical activity (71, 72) have been independently associated with an elevated risk of MS. There appears to be no difference between the sexes in association with MS and obesity in young adolescents (68). However, in adults, the association between obesity and MS appears stronger in females compared to males (73). Obesity leads to chronic inflammation which may contribute to MS pathology. Obesity has dramatically different consequences in males and females (74, 75); estrogen has been implicated as a central modulator in these pathways (76) whereas testosterone is likely beneficial in reducing adipose-induced inflammation (77). The relevance of these mechanisms for MS development remains unknown.

Vigorous exercise during adolescence is associated with a reduced risk of MS (71, 72). Long-term moderate physical activity reduces inflammation and risk of infection over time (78, 79) and its impact on the immune system may differ between male and females (80). However, how sex hormones contribute has been less investigated and there is little evidence to suggest that physical activity impacts the risk of MS differently in males and females (71).

### The Impact of Smoking and Nicotine on MS in Males and Females

Smoking increases the risk of developing MS, likely due to its inflammatory properties in the airways that translate into systemic and chronic inflammation (81). Smoking increases the risk of MS although one of the main components, nicotine, likely has protective effects (82). Once MS is established, smoking continues to contribute to MS pathology by increasing the rate of relapses and decreasing the time to progressive MS (83). Only a handful of studies have assessed the risk in males and females, with trends suggesting that historical smoking may increase the risk for MS more in males compared to females (82, 84). However, levels of nicotine metabolites in serum was more strongly associated with increased risk for MS in females suggesting instead that females may be more sensitive to smoking (85). That smoking has differential effects on males and females is long established (86) and recent studies also suggest that sex may be important in how smoking induces inflammation (86–88). Smoking may also impact sex hormone production and signaling (89). Increased rates of female smoking during the last decades, and possibly increased sensitivity to smoking metabolites, have been discussed as one of the reasons why MS has increased significantly faster in females compared to males over the last decades (85, 90).

### UVR and Vitamin D – Their Impact on MS in Males and Females

Decreased exposure to UV radiation (UVR) and vitamin D deficiency are independent risk factors for MS (91). Exposure to UVR modulates several aspects of the immune system (92, 93) and is essential for generation of active vitamin D. Vitamin D can also be obtained through diet and vitamin D itself has a range of immune modulatory properties that may be beneficial for reducing the risk of MS and other autoimmune diseases (94). The impacts of UVR and/or vitamin D on MS risk are interconnected and translate into a gradual increase in MS prevalence with increasing latitudes (95). However, the association between increased vitamin D levels and reduced risk of MS appears most prominent in fair-skinned individuals whereas increased UVR exposure is associated with reduced risk across several ethnic groups (96, 97). Furthermore, because vitamin D levels correlate with UVR exposure in fair-but not dark-skinned individuals, it has been argued that MS protection is mediated through UVR exposure (97). Nevertheless, vitamin D is protective in EAE models, with dependency on genetic background (98) and several studies suggest that intake of high-vitamin D foods reduces the risk of MS (99). Clinical trials with vitamin D supplementation have shown mixed results (100). Regarding sex-specific effects, vitamin D supplementation mainly protects female mice from EAE (101) and human *in vitro* studies suggest that vitamin D is more effective in reducing CD4^+^ T cell proliferation in female, compared to male MS patients. The ability of vitamin D to induce FoxP3^+^ Tregs may also be dependent on estrogen (102). How these findings translate into clinical efficacy remains unclear. Isolated studies have reported that vitamin D is only associated with reduced disease incidence and severity in females compared to males (103). However, this requires further investigation as other studies report no sex-specific effects of vitamin D (104), or indeed no clinical benefit at all (100).

The direct impact of UVR exposure on MS pathology through its immunomodulatory properties has been less investigated (105–107). In mice, UVR reduces the risk of EAE through a vitamin D-independent mechanism (108). The interaction of UVR exposure and sex is unclear. Isolated studies suggest that UVR exposure may decrease the risk of MS more in females compared to males (109), and that latitude may contribute to the increase in females with MS (7) whereas others have found no difference in sex ratios across latitudes (110). UVR exposure may influence the production of sex hormones (111, 112) which recently were associated with hormone-induced behavioral changes (112). Whether the impact of UVR exposure on reducing MS risk and pathology is mediated through sex hormones remains to be investigated. There are a range of mechanisms proposed for how UVR exposure impacts the immune system (113) including induction of regulatory T cells, modulation of skin resident DC and recruitment of anti-inflammatory monocytes. UVR-induced immunoregulatory molecules such as *cis*-urocanic acid and nitric oxide may also be involved (92, 93) as may RNA-release from damaged keratinocytes (114). Our lab has demonstrated that UVR impacts the abundance of circulating B cell subsets and activation of TNF-producing B cells in patients with early MS (106, 115), unfortunately these studies were not powered to investigate the impact of UVR in males and females separately. Although not uniformly found (116), UVR exposure may also increase systemic type I IFN signaling (107), which may be beneficial for reducing MS manifestations.

## Discussion

The impact of sex hormones on autoimmune disease is complex. Not all female-dominated autoimmune diseases improve during pregnancy or by supplemental sex hormones. A classic example is SLE which is ~8 times more common in females compared to males (117). The risk of a SLE flare during pregnancy appears increased (118) and hormone replacement therapy increases the risk of mild flares (119). However, contraception does not significantly impact disease activity (120). Both SLE and MS are associated with previous EBV infection, reduced physical activity, obesity, smoking and reduced levels of vitamin D (121–124). However, the impact of sex hormones and UVR exposure differs substantially (125). Whereas, estrogen likely is beneficial in MS, the impact on SLE is less clear. UVR exposure is also beneficial in MS but in SLE, UVR exposure leads to a significant increase in manifestations, including skin rashes, and sun exposure can trigger disease flares (126). Whether this relates to an increase in dying cells from UVR exposure or UVR-induced type I IFN signaling (127), remains to be clarified. Notably, type I IFN signaling was upregulated in several immune cell subsets in MS patients treated with UVR (107) suggesting this may be a common response to UVR exposure. Avoidance of sun exposure is likely also responsible for the low levels of vitamin D observed in SLE patients.

With a change in environmental exposures, particularly in terms of increased pollution, airway irritants and reduced exposure to UVR through enhanced indoor lifestyles, the prevalence of MS may continue to increase. If women are more sensitive to these environmental exposures, we may continue to observe a disproportional increase of MS in females and possibly other autoimmune diseases.

## Conclusion

The immunological impact of sex significantly contributes to an increased prevalence of autoimmune disease in females compared to males. However, the immunological mechanisms driving this development likely differ across diseases and may be related to both sex-specific genetic differences, immunological impact of sex hormones and sex-specific responses to environmental stimuli. The influence on disease initiation and progression may vary. Further investigations into the impact of genetic sex and sex hormones on disease mechanisms are central. Targeting sex hormones and sex-specific inflammatory pathways as a strategy to decrease inflammation in autoimmune and rheumatic disease is becoming more popular (128–130). As has been detailed in this review, sex-specific mechanisms likely contribute to autoimmune disease through several independent pathways, and mapping these will be essential to better treat autoimmune diseases in the future.

## Author Contributions

JL drafted the manuscript. ST, SG, and PHH contributed to specific sections and edited the manuscript. All authors contributed to the article and approved the submitted version.

## Funding

The authors would like to acknowledge funding support from Multiple Sclerosis WA.

## Conflict of Interest

The authors declare that the research was conducted in the absence of any commercial or financial relationships that could be construed as a potential conflict of interest.

## Publisher's Note

All claims expressed in this article are solely those of the authors and do not necessarily represent those of their affiliated organizations, or those of the publisher, the editors and the reviewers. Any product that may be evaluated in this article, or claim that may be made by its manufacturer, is not guaranteed or endorsed by the publisher.
